# A fast solution to NGS library preparation with low nanogram DNA input

**DOI:** 10.1186/1753-6561-6-S6-P26

**Published:** 2012-10-01

**Authors:** Pingfang Liu, Gregory JS Lohman, Eric Cantor, Bradley W Langhorst, Erbay Yigit, Lynne M Apone, Daniela B Munafo, Christine Sumner, Fiona J Stewart, Thomas C Evans, Nicole M Nichols, Eileen T Dimalanta, Theodore B Davis

**Affiliations:** 1 New England Biolabs, Ipswich, MA 01938, USA

## 

Next-generation sequencing (NGS) has significantly impacted human genetics, enabling a comprehensive characterization of human genome as well as better understanding of many genomic abnormalities. By delivering massive DNA sequences at unprecedented speed and cost, NGS promises to make personalized medicine a reality in the foreseeable future. To date, library construction with clinical samples has been a challenge, primarily due to the limited quantities of sample DNA available. To overcome this challenge, we have developed a fast library preparation method using novel NEBNext reagents and adaptors, including a new DNA polymerase that has been optimized to minimize GC bias. This method enables library construction from an amount of DNA as low as 5 ng, and can be used for both intact and fragmented DNA. Moreover, the workflow is compatible with multiple NGS platforms.

